# Deep brain stimulation induces sparse distributions of locally modulated neuronal activity

**DOI:** 10.1038/s41598-018-20428-8

**Published:** 2018-02-01

**Authors:** YiZi Xiao, Filippo Agnesi, Edward M. Bello, Simeng Zhang, Jerrold L. Vitek, Matthew D. Johnson

**Affiliations:** 1 Biomedical Engineering, University of Minnesota, Minneapolis, USA; 2 Neurology, University of Minnesota, Minneapolis, USA; 3 Institute for Translational Neuroscience, University of Minnesota, Minneapolis, USA

## Abstract

Deep brain stimulation (DBS) therapy is a potent tool for treating a range of brain disorders. High frequency stimulation (HFS) patterns used in DBS therapy are known to modulate neuronal spike rates and patterns in the stimulated nucleus; however, the spatial distribution of these modulated responses are not well understood. Computational models suggest that HFS modulates a volume of tissue spatially concentrated around the active electrode. Here, we tested this theory by investigating modulation of spike rates and patterns in non-human primate motor thalamus while stimulating the cerebellar-receiving area of motor thalamus, the primary DBS target for treating Essential Tremor. HFS inhibited spike activity in the majority of recorded cells, but increasing stimulation amplitude also shifted the response to a greater degree of spike pattern modulation. Modulated responses in both categories exhibited a sparse and long-range spatial distribution within motor thalamus, suggesting that stimulation preferentially affects afferent and efferent axonal processes traversing near the active electrode and that the resulting modulated volume strongly depends on the local connectome of these axonal processes. Such findings have important implications for current clinical efforts building predictive computational models of DBS therapy, developing directional DBS lead technology, and formulating closed-loop DBS strategies.

## Introduction

Deep brain stimulation therapies, which employ high-frequency electrical stimulation, are known to modulate both neuronal firing rates and firing patterns in the stimulated nucleus, which in turn can disrupt pathological oscillatory activity and create complex informational lesions^1–3^. Such modulation motifs have been fairly well-characterized in the subthalamic nucleus and globus pallidus, which are two prominent DBS targets for Parkinson’s disease, and where prominent inhibition and complex spike activity phase-locked to the stimulation pulse train have been reported^4–8^. Much less is known, however, about the cellular responses during HFS in the cerebellar-receiving area of motor thalamus, which is the primary DBS target for treating Essential Tremor (ET). There is also a general lack of knowledge on the whereabouts and distribution within various target nuclei of neurons whose firing activity has been modulated by DBS. Exploring these unknowns will enhance our collective capacity to design more targeted approaches to DBS therapies for a range of brain disorders.

Computational models have suggested that motor thalamic HFS regularizes thalamocortical neuron spike activity adjacent to the active electrode^9,10^. These stimulus-entrained activity patterns are thought to stem from a combination of regularizing ion channel dynamics and entrainment of synaptic signaling^11,12^ by driving cerebellothalamic, corticothalamic, reticular nucleus, and thalamic interneuron afferents^13–17^. However, the relative synaptic innervation of afferents on motor thalamic neurons and the degree to which HFS affects each of these afferents^18,19^ is not well understood. What is known from *in vivo* experiments is that HFS in the motor thalamus results in suppression of local oscillatory activity^20,21^, generation of robust glutamate release^22^, and accumulation of adenosine that can inhibit spike activity during stimulation^23,24^.

The spatial distributions of neuronal firing rate and firing pattern changes around a thalamic DBS lead also have not been thoroughly investigated. Studies in other brain regions have suggested a sparse and long-range distribution of neuronal modulation within the stimulated nucleus. For instance, HFS at a 10 µA amplitude in the human globus pallidus internus was reported to suppress neuronal activity 250–600 µm away from the microelectrode tip^6^, which is significantly greater than the predicted maximum modulated somatic distance based on first principles^25^. Such findings, as suggested in visual cortex^26^, may stem from directly eliciting action potentials within local axonal processes that in turn connect with somata located distal to the active electrode. Similarly, given the complex network of interconnected neuronal processes within the motor thalamus, stimulation-induced modulation is likely to occur in a distributed manner, but this has not been thoroughly studied. In this study, we investigated the spatial context of motor thalamus spike activity to HFS in two healthy non-human primates chronically implanted with DBS arrays in the nucleus ventralis posterior lateralis pars oralis (VPLo), which is the homologue of the human DBS target for treating ET.

## Materials and Methods

### Animals

Two female rhesus macaque monkeys (*macaca mulatta*, Subject K and Subject U) were used in this study. All procedures were approved by the Institutional Animal Care and Use Committee of the University of Minnesota and were carried out in accordance with United States Public Health Service policy on the humane care and use of laboratory animals. The animals were given environmental enrichment, provided with water *ad libitum*, and given a range of food options including fresh fruit and vegetables. All efforts were made to provide good care and alleviate any discomfort for the animals during the study. Pre-operative 7T MRI was acquired at the Center for Magnetic Resonance Research at the University of Minnesota using a passively shielded 7T magnet (Magnex Scientific) for both animals. During the imaging sessions, the animals were anesthetized with isoflurane and monitored for depth of anesthesia. T2-W images were acquired with a 2D turbo spin echo sequence at 0.5 mm isotropic resolution using a field of view (FOV) of 128 × 96 × 48mm^3,27^. In a separate procedure, under isoflurane anesthesia, each animal was surgically implanted with a titanium headpost (Gray Matter Research) and a cephalic chamber (Crist Instruments) oriented in the parasagittal plane with an off-vertical angle of 31.0° in Subject K and 38.5° in Subject U. Each animal also received a post-implant CT scan to plan the DBS lead implantation using *Monkey Cicerone*^28^.

### DBS implant procedure

**Figure 1:**
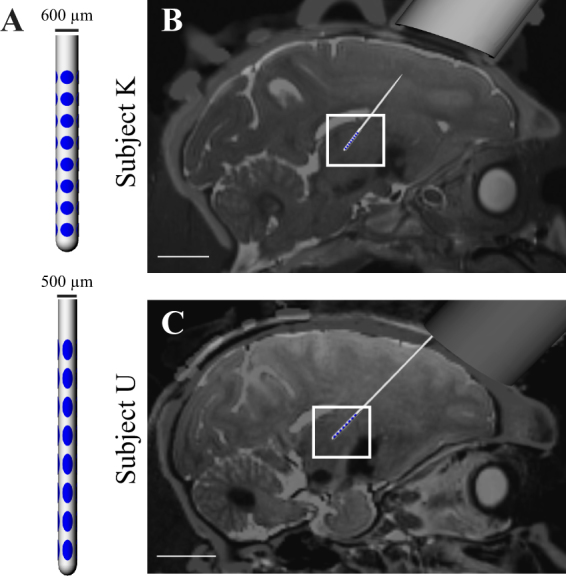
DBS Array Implants. (**A**) DBS arrays implantated in the cerebellar-receiving area of motor thalamus. (**B**,**C**) Co-registration of pre-operative T2-weighted MRI and post-implant CT images showing the final DBS array position and orientation in each subject. 1-cm scale bars are shown in (**B**,**C**).

Microelectrodes (250 µm shank diameter, 0.8–1.2 MΩ, FHC) were advanced through the chamber under the guidance of *Monkey Cicerone* to map the boundaries of VPLo. A combination of unit-spike responses to passive joint manipulation and microstimulation-evoked movements at thresholds less than 50 µA were used to identify VPLo and its borders^29^. The monopolar microstimulation parameters included a 0.5 second, 300 Hz train of biphasic, charge-based waveforms with a 100 μs cathodic phase, 20 μs interphase interval, and 100μs anodic phase. All monopolar stimulation settings applied for mapping and subsequent DBS experiments used a Gray Matter Research titanium headpost with bilaterally distributed titanium bone screw anchors over the parietal and occipital cranial regions as the return electrode. For each subject, the mapping track that yielded a long stretch of VPLo was chosen for chronic implantation of a DBS array. A radially segmented DBS array^30^ (NeuroNexus, Fig. 1A) with 32 ellipsoidal macroelectrodes (8 rows × 4 columns) arranged around a 600 µm diameter (Subject K) or 500 µm diameter shaft was chronically implanted into the VPLo through the pre-planned track. In Subject K, macroelectrodes had dimensions of 360 × 360 µm with 572 µm center-to-center distance between rows. In Subject U, macroelectrode sites were 280 µm wide × 530 µm tall with 750 µm center-to-center distance between rows. All electrodes were independent channels, electrically insulated from each other. Following DBS array implantation, a post-operative CT scan was performed to visualize the implantation trajectory, depth, and orientation of the DBS array using *Monkey Cicerone*. Each DBS array assembly had a flexPCB cable extending from the lead body within the cranial chamber. This assembly served as a fiducial marker for electrode column alignment along the lead shank. Prior to implantation, the DBS arrays were inspected under the microscope to confirm this alignment. The preoperative T2-W images were then co-registered with the postoperative CT to determine the DBS array position within the VPLo (Fig. 1B,C).

### DBS protocols

To visually assess the existence and in turn magnitude of stimulus-evoked motor twitches consistent with stimulation in the VPLo^29^, current controlled stimulation with parameters identical to those used during mapping was delivered through each electrode. The row of electrodes with the lowest threshold response for evoking a transient muscle contraction was chosen for stimulation and used in the microelectrode recording experiments in each animal (fourth row from the bottom in Subject K and the bottom row in Subject U). These muscle twitches were not sustained during longer duration stimulation trials. For subsequent recording experiments, a stimulus train at 100 Hz waveforms (charge-balanced and identical to those used for mapping) was delivered using a randomized presentation of three stimulus amplitudes (150, 250, and 350 µA) at each of the four electrodes along the selected row. At these stimulation frequencies and amplitudes, stimulus-induced muscle twitches were not observed and no motor deficits or other behavioral abnormalities were apparent. Current-controlled stimulation was used to reduce the propensity for *in vivo* impedance-based voltage fluctuations in tissue observed with constant voltage stimulation^31^. Current density at the stimulated electrode was 3.55 mA/mm^2^, which is higher than what has been used in ET patients (~0.39 mA/mm^2^)^32^, but is lower in overall peak current intensity (0.35 mA in this study, and 2.58 mA in humans, a factor of 7.4). This, however, must be put in context that the human cerebellar-receiving area of thalamus is approximately 8.2 times larger than the thalamic cerebellar-receiving area of in the rhesus macaque^33,34^. The tested amplitudes (150–350 *μA*) ensured electrode current densities also were within safe limits given the relatively smaller dimensions of the electrodes.

### Extracellular recordings

**Figure 2:**
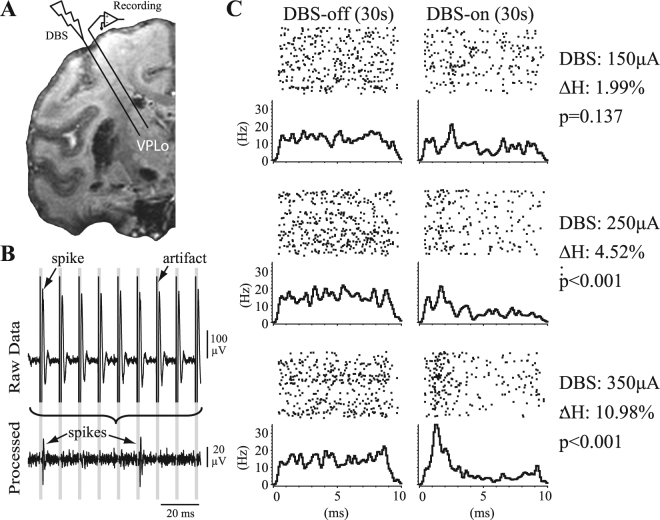
Single-unit spike recording analysis in the context of VPLo-HFS. (**A**) Microelectrode recordings were performed in the cerebellar receiving area of thalamus (VPLo) while stimulating in the same thalamic nucleus. (**B**) Recording artifacts were template-subtracted, with stimulation times used to generate peristimulus time histograms (PSTHs) of spike activity. Spike depolarization is shown as a negative polarity. (**C**) PSTHs from a recorded neuron before and during HFS at three different stimulation amplitudes. $\Delta {\boldsymbol{H}}$ is the percentage decrease in PSTH entropy between the HFS-off and HFS-on states. The ${\boldsymbol{p}}$ value indicates the likelihood of the observed PSTH entropy during HFS to occur by chance.

At least three weeks after DBS array implantation, stiff, epoxy dip-coated tungsten microelectrodes (250 µm shank diameter, 0.8–1.2MΩ, FHC) were inserted acutely around the chronically implanted DBS array in each animal (Fig. 2A). Wide-band recordings from these microelectrodes were digitized at 44 kHz (Alpha Omega SNR). Recordings were performed in the resting state before (30–60 s), during (60 s), and after HFS (30–60 s). Single-unit spike activity was recorded from 85 cells in Subject K (21 recording tracks) and 97 cells in Subject U (11 recording tracks). Extracting unit-spike activity involved filtering the data between 400–9000 Hz and removing stimulation artifacts using a template subtraction procedure^1,35^ (Fig. 2B). This process reduced the period of recording obscured by stimulation artifacts to a small blanked period (average ~0.5 ms). To prevent biasing the data, similarly blanked regions were introduced in the pre- and post-HFS recording epochs using “virtual stimulation” timestamps at the same stimulation pulse frequency. Template-subtracted spike recordings were thresholded and sorted in Offline Sorter (Plexon) to identify unit-spike activity and to verify that isolation of a single unit did not have spikes within a 2 ms refractory period. All recordings were selected based upon their good isolation characteristics through the spike sorting process. While most neuronal recordings were stable enough to record effects of HFS through each of the four radial electrodes at three different amplitudes (12 recordings in total), it was not possible to achieve this with every neuron (n = 135/182 were recorded across all configurations in both animals).

### Statistical Analyses

Time-stamps of spike activity, stimulation pulses, and virtual stimulation pulses were used to generate peri-stimulus time histograms (PSTHs, NeuroExplorer) (0.1 ms bins) before, during, and after HFS to visualize the degree of entrainment in spike activity to stimulation (or virtual stimulation) (Fig. 2C). PSTH comparisons between stimulation off/on conditions used the pre-HFS and HFS data, respectively, while visual inspection of the post-HFS data were used to verify that the unit-spike waveform shape and amplitude remained stable throughout the entire recording session. The post-HFS data will be reported in a follow-up study comparing neuronal activity within multiple DBS targets during the wash-out period after HFS. In terms of PSTH analysis, previous studies have used either thresholding^36^ or cumulative sum^37^ methods to determine instances of significant firing pattern modulation in PSTHs. However, these statistical methods do not quantify the gradual and often subtle changes in HFS-induced firing pattern modulation that are present within PSTHs. For this study, an entropy-based method was developed to quantify the degree of change in PSTHs between the off (immediately before HFS onset) and on (during HFS) states. Studies in the past have also used entropy as a quantitative measure of neuronal spike activity. However, the focus of these methods was to either measure neuronal information from inter-spike intervals^38–40^ or spike train data^41^. We considered entropy (H) of a PSTH and computed this variable using the following equation based on the frequentist interpretation^42^

1 $$H(x)=\,\sum _{i=1}^{m}p({x}_{i})log\frac{1}{p({x}_{i})}$$

where *m* is the number of bins in the PSTH, *x*_*i*_ is the total number of spikes that fell into PSTH bin *i* and $p({x}_{i})$ is the relative frequency that a spike falls into PSTH bin *i*. This formulation of PSTH entropy dictates that entropy will be high when spikes fall randomly within the inter-stimulus pulse period (i.e. a flat PSTH), and will be lower when stimulus-locked activity (i.e. peaks or troughs in the PSTH) occurs. The bin size in equation (1) was chosen to be 0.5 ms, consistent with the shortest observed interval of entrained spike responses in the PSTH when considering a very narrow bin size. Bin sizes that were too narrow or too broad deprived equation (1) of the power to capture a decrease in H. The first 0.5 ms in each PSTH was excluded from analysis to avoid false positives related to the blanking period from the stimulus subtraction algorithm. Excitatory or inhibitory firing pattern modulation manifested as a drop in H between HFS-off and HFS-on periods as calculated by:

2 $${\rm{\Delta }}H \% \,=\,\frac{{H}_{HFS\_off}-\,{H}_{HFS\_on}\,}{{H}_{HFS\_off}}\times 100 \% $$

Statistical significance of firing pattern modulation was determined by sampling with replacement *n* spikes from the HFS-off period (where *n* equals the total number of spikes in the HFS-on period); calculating H using equation (1); repeating this process 10,000 times to generate a bootstrapped distribution of HFS-off PSTH entropies (${H}_{HFS\_off}$); and, computing the HFS-on PSTH entropy (${H}_{HFS\_on}$). Statistically significant firing pattern modulation was defined by cases when the bootstrapped ${H}_{HFS\_off}$ distribution was less than ${H}_{HFS\_on}$ with a probability less than 5% (α = 0.05) (Fig. 2C). Firing rates calculated before, dur_ing, and after HFS were also compared for each recorded cell. A statistically significant difference in firing rate was established using the Mann-Whitney U test (1 s bins, *p* < 0.01). Significant excitatory firing pattern modulation or firing rate increase were designated as ‘p+’ and ‘r+’, respectively, while significant inhibitory firing pattern modulation or firing rate decrease were designated as ‘p−’ and ‘r−’. No significant change in any of the above categories was designated as ‘n’. A small fraction of recordings (<2%) were observed to have intermittent and very sparse spike activity (<1 Hz) in either the HFS-off or HFS-on periods, which led to inaccurate representations of the PSTH entropy. These recordings were excluded from further analysis. The datasets generated and analyzed during the current study are available from the corresponding author on reasonable request.

To contextualize the spatial distribution of the PSTH results, two methods were used. The first involved charting the putative locations of microelectrode recordings defined through the microdrive relative to the CT localization of the DBS array. The Narishige microdrive, used for the microelectrode recording experiments, provided a snug fit for the insertion guide tube to puncture the trajectory of the microelectrode during insertion. Coordinates for implantation were determined using the Narishige x-y positioner, which had an x-y spatial resolution of 100 µm and depth resolution of 10 µm. In the second case, the size of the monopolar stimulus artifact recorded from the microelectrode was used as a proportional measure of distance, according to volume conductor theory^43,44^. For each recording session, the artifacts from stimulation amplitudes (150, 250, or 350 µA) were each averaged, and the peak magnitude (mV) of the recorded waveform was used as the pseudo measure for distance. Neuronal recordings were then assigned based on observations that neurons generally need to be within 50 *μm* of a microelectrode in order to be reliably identified in the spike sorting process^45,46^.

## Results

### Heterogeneity of Neuronal Responses to HFS in the VPLo

**Figure 3:**
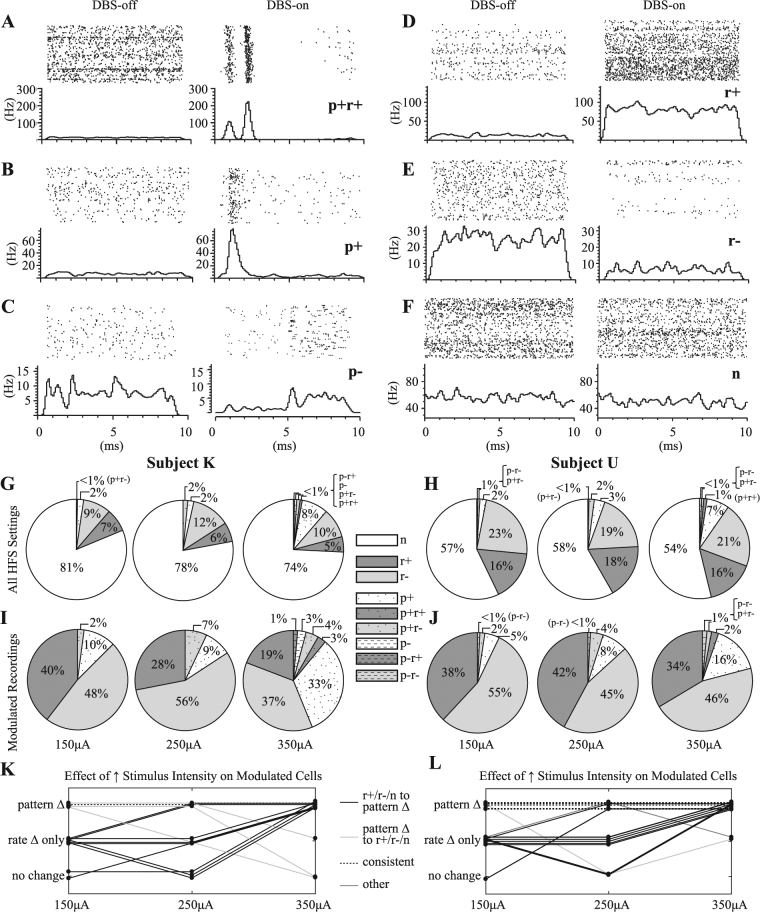
Heterogeneity of thalamic PSTH responses to VPLo-HFS with increasing stimulation amplitude. (**A**–**F**) Representative examples of the following classes of responses (p: firing pattern modulation, r: firing rate modulation. ‘+’: increase in firing rate or phase-locked spike activity, ‘−’: decrease in firing rate or phase-locked spike activity, n: no response). (**G–J**) Dependence of stimulation amplitude on motor thalamic neuronal responses to VPLo-HFS. Data from Subject K and Subject U are on the left and right side of the legend, respectively. The top row shows the proportionate effects of HFS at 150, 250, and 350 ${\boldsymbol{\mu }}A$ across all recordings (Subject K: n = 245, Subject U: n = 283), whereas the bottom row shows the corresponding percentages only for those recordings that were significantly modulated by HFS (i.e. firing pattern or rate modulation). (**K,L**) Effect of stimulus amplitude on all cells exhibiting one or more instances of firing pattern modulation (n = 11 cells in Subject K, n = 21 cells in Subject U).

Thalamic responses to HFS exhibited firing rate and/or firing pattern modulation in both subjects (Fig. 3A–F). A total of 65.5% (54/85) and 74.2% (72/97) of the recorded cells in Subject K and Subject U, respectively, showed exclusively firing rate modulation for one or more of the stimulation amplitudes and electrode configurations (up to 4 electrodes × 3 amplitudes = 12 instances of HFS for a given cell). Additionally, 12.9% (11/85) and 21.7% (21/97) of the recorded cells in Subject K and Subject U, respectively, exhibited firing pattern modulation (p+ or p−) that may or may not have also included firing rate modulation (e.g. p+r+, p+r−, p−r+, or p−r−) during HFS.

The recordings were subsequently grouped by stimulation amplitude and divided into nine categories that included spike activity phase-locked to the stimulus pulse with either excitatory or inhibitory entrainment as well as overall changes in firing rate with both excitatory and inhibitory effects (Fig. 3G–J). Of the recordings that exhibited modulation of spike activity, the percentage of responsive recordings increased modestly from 19% at 150 µA to 26% at 350 µA in Subject K and from 43% at 150 µA to 46% at 350 µA in Subject U. More notably, however, for recordings showing modulation to HFS, there was a shift from primarily firing rate modulation to a more diverse collection of firing rate and firing pattern responses. More specifically, ‘r+’ and ‘r−’ responses accounted for 88% and 56% of all responsive recordings at 150 and 350 µA, respectively, in Subject K. Similarly, the proportion of firing rate modulation responses was 94% at 150 µA and 80% at 350 µA in Subject U. This decrease was balanced by an increase in the proportion of firing pattern modulation responses in that the majority of cells exhibiting firing pattern modulation at any of the stimulus amplitudes transitioned from no modulation or only rate modulation at lower stimulus amplitudes to pattern modulation with or without rate modulation at higher stimulus amplitudes (Fig. 3K,L).

### Neuronal Pattern Responses as a Function of Stimulus Amplitude

The strength of firing pattern modulation, as a function of stimulus amplitude, was further investigated. Only those recording sessions that underwent HFS at all three amplitudes (150, 250, and 350 µA) were included in this analysis. The recordings were grouped into two categories: HFS failed to elicit firing pattern modulation (i.e. ‘n’, ‘r+’ and ‘r−’) at any of the three stimulation amplitudes (Group nFPM), and HFS elicited firing pattern modulation (i.e. ‘p+’, ‘p−’, ‘p+r+’, ‘p+r−’, ‘p−r+’ and ‘p−r−’) with at least one stimulation amplitude (Group FPM). Subject K had 224 and 21 recordings in Groups nFPM and FPM, respectively. Subject U had 251 and 32 recordings in Groups nFPM and FPM, respectively.

**Figure 4:**
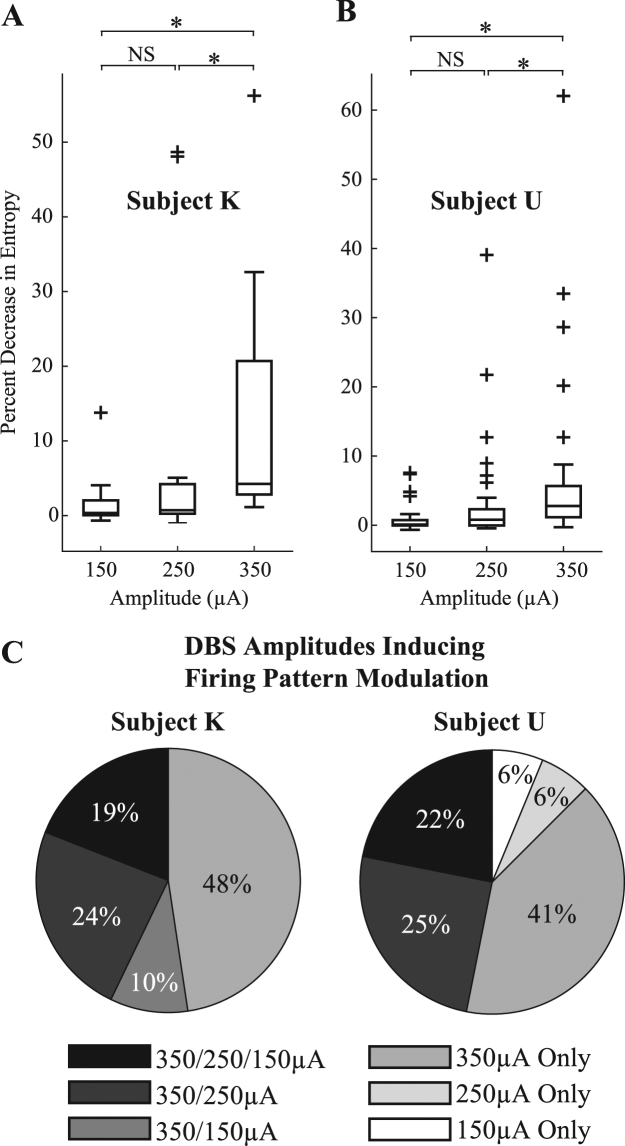
Effect of HFS amplitude on PSTH entropy change and firing pattern modulation. (**A,B**) Shown are population-based entropy changes in those cells with at least one significant entropy change amongst the three stimulus amplitudes. NS: no significant difference by multiple comparison test. *Significant difference by Mann-Whitney U test with Bonferroni correction (α = 0.0167, FPM cells: Subject K, n = 21 recordings; Subject U, n = 32 recordings). (**C**) Likelihood of firing pattern modulation to occur across the three different stimulus amplitudes.

Within each group, one-way ANOVA was performed to test for a significant difference (α = 0.05) in the average ${\rm{\Delta }}H \%$ with stimulus amplitude as the explanatory variable. A significant difference was found in Group FPM for both animals (Subject K: p = 0.028, Subject U: p = 0.021), but not Group nFPM for both animals (Subject K: p = 0.276, Subject U: p = 0.247). Multiple-comparison tests (n = 3, Mann-Whitney U test with Bonferroni correction, α = 0.05/3 = 0.0167) were subsequently performed on the data in Group FPM. In both animals, significant differences were found between the 150–350 µA groups and the 250–350 µA groups, but not between the 150–250 µA groups (Fig. 4A,B). The analysis also showed that firing pattern modulation was more likely to occur at higher stimulus amplitudes, and more specifically, the 350 µA case, which accounted for 48% and 41% of all recordings with firing pattern modulation in Subject K and U, respectively (Fig. 4C).

### Distribution of Neuronal Firing Pattern Modulation around the DBS Array

**Figure 5:**
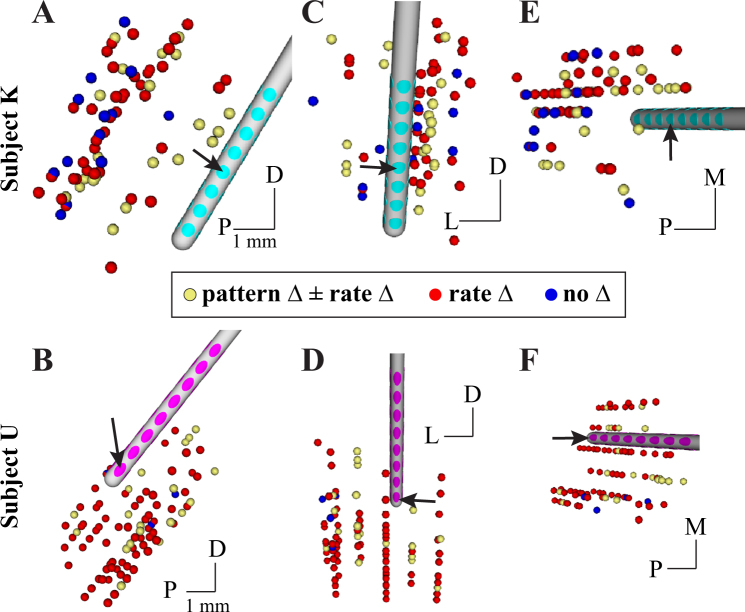
Distribution of modulated motor thalamic activity with respect to microdrive-defined recording site location. Recording location sphere colors correspond to whether no modulation was observed for any stimulus setting (blue), only a change in firing rate was observed (red), and if a firing pattern change was observed regardless of whether or not a rate change was also observed (yellow). Perspectives are shown for sagittal (**A**,**B**) coronal (**C**,**D**) and axial (**E**,**F**) perspectives in both subjects. D: dorsal; P: posterior; L: lateral; M: medial. Scale bars indicates 1-mm.

The spatial distribution of modulated activity within motor thalamus was investigated in two contexts (Fig. 5). First, recording site locations were estimated generally from co-registration of the microdrive coordinate system with the post-operative CT scan, which showed both microdrive and array implant positions and orientations. For this spatial analysis, recording site locations were considered to have a firing pattern change if any of the stimulation amplitudes or electrode configurations induced a statistically significant firing pattern change. Most recording site locations exhibited only firing rate modulation with locations both proximal and distal to the row of active electrodes. Recording site locations with no modulation occurred at more distal locations relative to the active electrodes. Recording site locations in which firing pattern modulation, with or without firing rate modulation, were also distributed around and along the DBS array. Notably, recording site locations immediately adjacent to one another were found to exhibit firing rate modulation but no firing pattern modulation, and vice versa.

**Figure 6:**
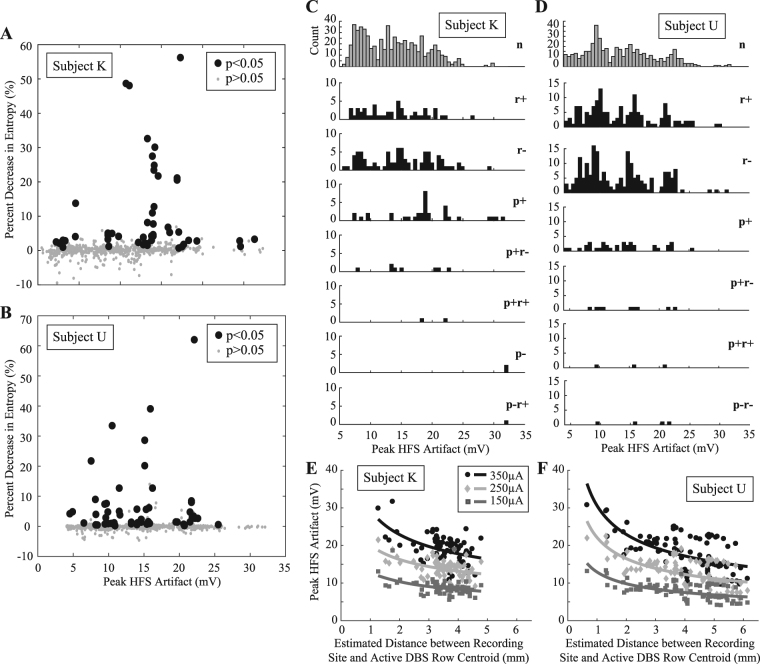
Distribution of modulated motor thalamic activity with respect to stimulation artifact size. Percent decrease in PSTH entropy between the HFS-off and HFS-on periods plotted against the peak amplitude of the recorded HFS artifact for (**A**) Subject K and (**B**) Subject U. Recordings that exhibited significant firing pattern modulation during HFS are labeled as large black solid circles. (**C**,**D**) Spatial distribution of neuronal recordings grouped by their response to HFS (p: firing pattern modulation, r: firing rate modulation. ‘+’: increase in firing rate or phase-locked spike activity, ‘−’: decrease in firing rate or phase-locked spike activity, n: no response). (**E**,**F**) Relationship between the stimulus artifact peak amplitude and the estimated recording site distance from the centroid point of the active DBS row. Shown are data for both subjects categorized based on stimulus amplitude (150, 250, and 350 µA).

Additionally, the average peak stimulus artifact amplitude was used as a pseudo-measure of distance between the active stimulating electrode and the microelectrode recording the neuronal spike activity. Stimulus artifact amplitude was related to stimulus-induced firing pattern changes in a total of 783 and 872 recordings in Subject K and Subject U, respectively, with each recording corresponding to HFS with a single stimulus amplitude. PSTHs were created for each recording, and the ${\rm{\Delta }}{\bf{H}}{\boldsymbol{ \% }}$ between the HFS-off and HFS-on epochs was calculated and plotted against the peak stimulus artifact amplitude (Fig. 6A,B). Similar to the microdrive-based calculations, a distributed and sparsely populated spatial profile of firing pattern modulation was observed. Instances of significant firing pattern modulation occurred over a wide range of stimulus artifact amplitudes and changes in PSTH entropy. The data also showed that recordings with either firing rate or firing pattern modulation responses to HFS had spatially distributed profiles in both subjects without a clear difference in the median stimulation artifact amplitude (Fig. 6C,D). The relationship between the stimulus artifact amplitude and the estimated recording site distance (d) was consistent with a nonlinear decay function that was proportional to 1/d^x^ (Fig. 6E,F). Nonlinear curve fitting resulted in estimates for x as 0.36, 0.31, and 0.32 for the 350, 250, and 150 µA stimulus intensities in Subject K, respectively, and for x as 0.42, 0.42, and 0.40 for the 350, 250, and 150 µA stimulus intensities in Subject U, respectively. The coefficient of determination (R^2^) for these curve fits ranged between 0.17–0.23 for Subject K and between 0.33–0.36 for Subject U.

## Discussion

The results of this study indicate that there is substantial heterogeneity in local neuronal responses to motor thalamic HFS, including modulation of firing rate and pattern across populations of neurons within motor thalamus. Further, the volume of tissue modulated in terms of firing pattern around the implanted DBS array was sparsely populated and not confined to the immediate vicinity of the active electrode. Increasing stimulation amplitudes induced both a greater change in the strength of the firing pattern entropy difference as well as recruited a denser population of motor thalamic neurons within the volume of tissue modulated. Previous studies have mostly characterized thalamic neuronal responses to short duration intrathalamic stimulation^12,29,47–49^ with fewer studies investigating intrathalamic effects on the order of 10 s of seconds^50,51^. The data presented in this study also expand on the spatiotemporal changes in neuronal spike activity that occur during longer duration HFS within the motor thalamus.

HFS induced primarily firing rate modulation and in particular a decreased spike rate without complete cessation of spike activity *during* HFS. These findings extend previous reports showing that the major effect *following* short periods of thalamic stimulation was local depression of neuronal activity characterized by complete silencing of neuronal spike activity^52^ or more complex burst patterns followed by quiescent periods that depended on stimulation parameters^12^. Firing pattern modulation was observed less frequently with ‘p+’ type responses being most common in both subjects. The relative difference in firing rate versus pattern modulation may stem in part from the prevalence or strength of excitatory relative to inhibitory inputs within VPLo thalamus^10,11,16^. Moreover, the results may also stem from the ability of HFS to more readily modulate specific axonal afferent and efferent processes based upon the relative orientation of the electric field to the axonal trajectories within motor thalamus^53^.

The majority of recordings showed that neuronal activity was not widely influenced by all HFS settings. These findings are consistent with previous reports, where 55%^47^ and 51%^49^ of motor thalamic neurons were unaffected by stimulation following brief (0.5 ms) periods of stimulation with amplitudes ranging from 120–200 µA. It is important to note, however, that increasing stimulation amplitude in our study was found to result in greater firing pattern modulation at the expense of firing rate modulation alone. For cells exhibiting firing pattern modulation at any one of the stimulation amplitudes, the results showed a stronger degree of firing pattern modulation for a given cell with increasing stimulation amplitude. This difference in modulatory effect was found between 250–350 µA and not between 150–250 µA, suggesting a nonlinear recruitment effect with stimulation amplitude. Together, these results suggest that additional increases in stimulation amplitude may further shift the population responses and the strength of the modulatory effect from one primarily based on firing rate modulation to one showing a broad class of firing rate and pattern modulation within motor thalamus. The clinical implication for these results is that, at lower stimulation amplitudes, the mechanism of ‘de-rhythmication’^51^ may involve ‘masking’ rhythmic tremor activity within thalamus^54^ by creating noise in the population spike activity (for firing rate increases) or by raising the threshold for propagation of tremor burst activity (for firing rate decreases). At higher stimulus amplitudes, however, the results indicate that the mechanism shifts to disruption of tremor activity within thalamus via firing pattern modulation of spike activity, which is known to induce information lesions^1^.

The recording data also showed that instances of significant firing rate and pattern modulation occurred over a wide range of distances that were not confined to the immediate vicinity of the active electrode. This resulted in neuronal response subtypes for any given stimulation setting being sparsely distributed around the DBS array. This sparse distribution of modulated neurons is consistent with observations from other studies. Following brief stimulation in the ventrolateral nucleus (VPLo homologue) in cats, non-responsive cells were reported to be widely distributed, even very close (<1 mm) to the stimulating electrode^52^. Similar sparse and distributed spatial modulation profiles have also been observed in the visual cortex using two-photon calcium imaging^26^. Interestingly, the variation in the ${\rm{\Delta }}$H% observed amongst the modulated cells that we observed in our study (Fig. 6) may also represent the underlying phenomenon visualized with calcium imaging as a difference in fluorescence intensity of activated cells^26^. One explanation for both the heterogeneous responses and the distributed spatial modulation profiles is that electrical stimulation results in very local excitation of axons, which pass adjacent to the active electrode. The afferents and efferents within motor thalamus are known to have diverse branching patterns and trajectories^55^, and driving action potentials within these axons would result in a complex set of antidromic and orthodromic responses within motor thalamus consistent with the observations from this study.

There are several considerations in interpretation of the results, including that the data were not collected in a disease model. Harmaline treatment is the most established model to induce action tremor^56^; however, harmaline-induced tremor is transient and studies have shown that animals develop resistance to the treatment^57,58^ making it impractical for the experimental protocols conducted in this study. Future studies that leverage multi-channel electrode arrays in conjunction with harmaline treatment and a movement task will be important to validate the results of this study in the context of action tremor. Another study limitation is the size of the stimulation artifact as a pseudo-measure of distance between recorded neurons to the stimulating electrode. This approach was implemented because exact Euclidian distances were not readily available from histology in the two subjects. However, the relationship between the peak stimulus artifact amplitude and the estimated distance from the corresponding recording site location and the row of active DBS showed a nonlinear decay function that was consistent with that reported previously^43^. Variation in the curve fitting could stem from slight discrepancies in the estimated site locations from the microdrive coordinate system as well as shadowing that may occur between electrodes active on one side of the DBS lead and recording sites performed on the other side of the DBS lead. Finally, the range of stimulation amplitudes investigated in this study was relatively small in scope. However, the data point towards larger stimulation amplitudes further increasing the percentage of cells modulated and further increasing the percentage of responses identified as firing pattern modulation.

As far as we know, this study is the first of a kind effort to map the distribution of various HFS-induced changes in neuronal activity around a chronically implanted DBS array. Motor thalamus unit-spike recordings exhibited several classes of firing rate and firing pattern responses to locally-applied HFS with broad spatial distributions. Increasing stimulation amplitude was found to non-linearly increase the modulation volume density but also shift the type of modulation from one based primarily on firing rate modulation to one based in greater measure on firing pattern modulation. Together, these mechanistic findings have important implications for computational models of DBS^11,59–62^, closed-loop stimulation therapies^62^, as well as development of targeting approaches to more selectively modulate deep brain cellular pathways^11,59,61,63^.
